# Three discipline collaborative radiation therapy (3DCRT) special debate: We should treat all cancer patients with hypofractionation

**DOI:** 10.1002/acm2.12954

**Published:** 2020-06-29

**Authors:** Michael Green, Samantha J. Van Nest, Emilie Soisson, Kathryn Huber, Yixiang Liao, William McBride, Michael M. Dominello, Jay Burmeister, Michael C. Joiner

**Affiliations:** ^1^ Department of Radiation Oncology University of Michigan Ann Arbor MI USA; ^2^ Department of Radiation Oncology Weill Cornell Medicine New York NY USA; ^3^ Department of Radiology University of Vermont Burlington VT USA; ^4^ Department of Radiation Oncology Tufts Medical Center Boston MA USA; ^5^ Department of Radiation Oncology Rush University Medical Center Chicago IL USA; ^6^ Department of Radiation Oncology University of California at Los Angeles (UCLA) Los Angeles CA USA; ^7^ Department of Oncology Wayne State University School of Medicine Detroit MI USA; ^8^ Gershenson Radiation Oncology Center Barbara Ann Karmanos Cancer Institute Detroit MI USA

## 1 Three Discipline Collaborative Radiation Therapy (3DCRT) Debate Series

Radiation Oncology is a highly multidisciplinary medical specialty, drawing significantly from three scientific disciplines — medicine, physics, and biology. As a result, discussion of controversies or changes in practice within radiation oncology must involve input from all three disciplines. For this reason, significant effort has been expended recently to foster collaborative multidisciplinary research in radiation oncology, with substantial demonstrated benefit.^1^, ^2^, ^3^ In light of these results, we endeavor here to adopt this “team‐science” approach to the traditional debates featured in this journal. This article is part of a series of special debates entitled “Three Discipline Collaborative Radiation Therapy (3DCRT)” in which each debate team will include a radiation oncologist, medical physicist, and radiobiologist. We hope that this format will not only be engaging for the readership but will also foster further collaboration in the science and clinical practice of radiation oncology.

## 2 Introduction

Better Physics, just within this 21st century, now enables us to deliver radiation to a target volume with accuracy better than 1 mm. Given this accuracy, why fractionate at all? If we can put dose only on the cancer, and extremely little on critical normal tissue, then surely just give a high single dose to that cancer, and job done. Local tumor control is 100% with minimal toxicity. If only. Two linked issues keep Biology (radiobiology) in the clinical game. First, our ability to identify, localize, and immobilize anatomy and pathology does not yet correspond with this submillimeter accuracy of radiotherapy delivery. Second, even if that imaging resolution is reached it could still not detect occult disease. Consequently, unless the cancer is truly isolated, which it sometimes may be, for example, in organ‐confined early‐stage prostate cancer, it is always necessary to “degrade” the treatment plan by defining a CTV and PTV into which the radiation delivery is expanded. This inevitably imposes a risk of normal‐tissue radiotoxicity, therefore we must use fractionation to minimize that risk. Traditionally, that fractionation has been carried out with doses close to 2 Gy per fraction. In fractionation, the Linear‐Quadratic (LQ) model describes the relationship between total dose and dose per fraction, for isoeffect. A lower *α/β* value indicates a steeper relationship. Generally late‐reacting normal tissues exhibit lower *α/β* and early‐reacting normal tissues exhibit higher *α/β*. Malignancies can have lower or higher *α/β* depending on the tumor type. In some malignancies, notably human prostate, clinical data indeed indicate *α/β* as low as 1.5 and thus in prostate cancers, and likewise in early‐stage breast cancers, hypofractionation, arbitrarily defined as a dose per fraction> 2.2 Gy, has become standard of care. In early‐stage non‐small cell lung cancers a higher *α/β* is seen, similar to early‐reacting normal tissue, but these isolated malignancies can still be more effectively controlled with radical hypofractionation which suggests the story is more complex than a simple LQ picture can paint. So are we moving, and should we be moving, even if slowly but surely, toward giving all patients receiving radiation therapy as hypofractionation? Let us debate!

Arguing for the proposition will be Drs. Michael Green, Samantha Van Nest, and Emilie Soisson. Dr. Green is an Assistant Professor at the University of Michigan in the Department of Radiation Oncology. His group utilizes expertise in quantitative immunophenotyping, peripheral tolerance mechanisms, and tumor cell death to define and harness the determinants of inflammation, which shape antitumoral immunity and influence radiotherapy and immunotherapy efficacy. Dr. Van Nest is a Postdoctoral Associate in the Department of Radiation Oncology at Weill Cornell Medicine in New York. Dr. Van Nest's research focuses on developing techniques for the personalization of radiation therapy including Raman spectroscopy and omics‐based signatures. She is investigating mechanisms of radiation‐induced anticancer immunity with particular focus on patient‐based platforms for optimizing immune activation. Dr Soisson is a medical physicist at the University of Vermont Medical Center. She holds faculty appointments at the University of Vermont and McGill University. She earned a PhD in Medical Physics at the University of Wisconsin where she was heavily involved in the clinical implementation of TomoTherapy.

Arguing against the proposition will be Drs. Kathryn Huber, Yixiang Liao, and William McBride. Dr. Huber is a Radiation Oncologist at Tufts Medical Center and Assistant Professor at Tufts University School of Medicine, Boston, MA. She specializes in the treatment of thoracic, breast, and head and neck cancers and is the Director of Radiobiology for the residency training program at Tufts. Dr. Liao is a faculty medical physicist at the Rush University Medical Center, Chicago, IL with research interests in brachytherapy, IGRT, SBRT, and treatment planning. Her publications cover various topics including hypofractionation in prostate cancer treatment, and normal tissue tolerance in treatments delivering high dose per fraction such as high‐dose rate (HDR) brachytherapy and intraoperative radiation. She also currently serves as the associate director of the medical physics residency program at Rush. Dr. Liao was among the students of the first IBPRO^1^ (Integrated course in Biology and Physics of Radiation Oncology) held at Wayne State School of Medicine in 2014. Dr. McBride is emeritus professor and former Vice‐Chair for Research in the Division of Cellular and Molecular Oncology, Department of Radiation Oncology at UCLA. Most of his extensive research has woven immunological concepts into understanding of radiation responses by normal tissues and tumors, in particular the response of the immune system as it senses “danger” signals from radiation‐damaged tissues. Dr. McBride has been honored with an ASTRO Gold Medal and Failla Award from the RRS.

## 3 Opening Statements

### 3.A Michael Green, MD, PhD; Samantha Van Nest, PhD; Emilie Soisson, PhD (FOR)

Hypofractionated radiotherapy is characterized by the delivery of greater than 2.2 gray (Gy) per fraction and a reduced number of fractions as compared to conventional fractionation. At the turn of the century, now classic radiobiologic experiments showed that fractionated radiotherapy increased tumor control while diminishing acute toxicity. These empirical observations led to the development of one of our fields' most dear beliefs: that fractionated radiotherapy delivered in 1.8 to 2.0 Gy per day is the most efficacious and safest way of delivering radiotherapy.^4^ In the past decades, transformative improvements in immobilization, target and normal tissue delineation with multiple imaging modalities, treatment planning, and patient alignment have been made. These advances have led to unprecedented potential for safe and effective delivery of high‐dose radiotherapy. This has led to the thoughtful and timely re‐evaluation of how we provide definitive management and a questioning of our core principles regarding fractionation. The conclusion of this re‐evaluation is clear: we should treat all cancer patients with hypofractionation.

Multiple randomized trials have suggested that hypofractionated radiotherapy provides superior outcomes in both the definitive and palliative setting. The CHISEL trial demonstrated that ultrahypofractionated radiotherapy was superior to conventional fractionation for Stage I NSCLC.^5^ Furthermore, ultrahypofractionated approaches have now been shown to provide more durable palliation as compared to standard palliative regimens.^6^ Moderate hypofractionation has proved noninferior in randomized phase III trials for localized prostate^7^, ^8^, ^9^, ^10^ and breast cancer.^11^ Long follow‐up of these trials as well as meta‐analyses have not demonstrated the hypothesized increase in late toxicity with hypofractionated regimens.^12^, ^13^, ^14^ Together, these data support hypofractionation in the management of many diseases.

Hypofractionation has only been made possible by improvements in tumor localization through advances in image‐guided radiation therapy (IGRT). Traditional stereotactic localization, which relied on external targeting frames and specialized equipment, has been replaced by volumetric image guidance (CT, CBCT, MVCT) and stereoscopic imaging which have become accessories on standard accelerators and are now generally available in all clinics. In addition, a wide variety of motion management strategies are routinely employed to quantify, measure, and track intrafraction tumor motion. Daily 3D alignment and motion management have provided an opportunity to explore dose escalation through reduced tumor margins and moderate hypofractionation in sites where tumor dose was previously limited by normal tissue tolerances. Intriguingly, margins have not been shrinking as much as they could be based on localization ability alone. Numerous studies have shown improved geometric accuracy is achievable with IGRT which should go hand in hand with reduced margins, but surveys of practice patterns showed no relationship between the frequency of image guidance and PTV margins.^15^ Practice change as a result of improved technology is lagging and future clinical trials are still needed to realize the potential of improved localization.

The superiority of conventional fractionation is supported by linear‐quadratic (LQ) modeling of radiation effects. These models assume that the alpha/beta (*α/β*) value in tumor is higher than that of the surrounding normal tissue, but re‐evaluations have found some tumors actually have an *α/β* value lower than normal tumors.^16^, ^17^ Moreover, the LQ model can break down at the doses used in ultrahypofractionation.^18^ AAPM has initiated efforts to revise the LQ model (Hypofractionation Treatment Effects in the Clinic, www.aapm.org/pubs/hytec). Thus, prior theoretical concerns have given way to the rapid study and clinical adoption of hypofractionation.

The universal benefit of hypofractionation (and possible source of breakdown of the LQ model) rests in the potential role of novel radiobiological factors that may become relevant at higher doses per fraction. Hypofractionation challenges the long‐held understanding of how RT controls tumors, redefining its role in immune activation, reoxygenation, and repopulation. Hypofractionation is particularly effective at inducing immunogenic cell death (ICD)^19^, ^20^ leading to antitumor immunity.^21^, ^22^ In contrast, conventional fractionation promotes more immunosuppressive cell death pathways.^23^, ^24^ Dose‐dependent increases in tumor antigen presentation further improve opportunity for antitumor immunity with hypofractionation.^24^, ^25^

Evidence suggests the inflammatory effects of RT are activated only above a threshold dose of around 6‐8 Gy^26^, ^27^ leading to increased local secretion of key cytokines, chemokines, and other molecules that initiate and support an adaptive immune response.^28^ Furthermore, the steep dose falloff typical in hypofractionated regimens may establish a cytokine gradient that promotes increased tumor infiltrating lymphocytes (TILs).^28^, ^29^ Hypofractionation has been shown to enhance TILs,^30^ increase DC recruitment/maturation,^19^, ^29^, ^31^ and activates peripheral CD8^+^ T cells in several cancer models.^20^, ^32^, ^33^ Tumors with increased levels of active lymphocytes may benefit from increased spacing between fractions, which can be facilitated by hypofractionation.^30^, ^33^, ^34^

The immunogenic effects of hypofractionation can be further exploited through combination with immune checkpoint blockade (ICB). Hypofractionation has been recommended as the optimal strategy for combination with ICB,^35^ showing systemic tumor reduction and abscopal response at the preclinical and clinical level in a variety of tumor types.^34^, ^35^, ^36^, ^37^, ^38^, ^39^, ^40^, ^41^, ^42^ Hypofractionation may enhance antitumor immunity by increasing neoantigen exposure and broadening the T‐cell receptor repertoire.^27^, ^33^, ^43^, ^44^ These findings support the adoption of hypofractionation to improve immune stimulation and potentially induce an abscopal response.^24^

In addition to promoting immune infiltration, hypofractionation can modulate tumor vasculature.^24^, ^45^ Higher doses per fraction (5–10 Gy) applied more than once have been shown to cause reduced blood flow to the tumor and vascular deterioration.^46^ While some reoxygenation may occur following hypofractionation, the hypoxic fraction is further reduced through ischemic cell death to a subpopulation that would have been inherently radioresistant.^47^

Hypofractionation also reduces the time to achieve the prescribed dose, possibly countering the role of accelerated tumor repopulation during later stages of RT.^48^, ^49^, ^50^ A shortened course of therapy is not only beneficial from a radiobiological perspective but also it provides significant cost savings, both to the providers and patients.^51^, ^52^ It is increasingly recognized that protracted work interruption contributes significantly to the accumulation of medical debt and a rise in bankruptcy.^53^

As we seek to improve the quality of care we provide, we must remember that hypofractionation offers effective patient‐centered high‐value care. Randomized clinical trials, physics advances, and new radiobiological paradigms point to a new simple truth: all patients should receive hypofractionated radiotherapy.

### 3.B Kathryn Huber, MD, PhD; Yixiang Liao, PhD; William McBride, PhD, DSc (AGAINST)

Received wisdom from over a century of radiation treatment of cancer is that dose is best delivered fractionated in a long course over typically 5–7 weeks. Historically, attempts to use hypofractionation to condense treatment time into a more convenient and cost‐effective scale gave generally inferior outcomes. As a result, radiation oncologists came to associate high dose per fraction with more severe side effects. Clinical experience was mirrored by radiobiological experiments showing the importance of tissue growth kinetics in modulating effects of changing size of dose per fraction and overall treatment time.^54^ In brief, fractionation spares tissues with slow turnover relatively more than “acute” tissues that are capable of rapid renewal, although these are spared by proliferating during long‐course treatment. In other words, as dose per fraction decreases, the isoeffective total dose for an effect increases more rapidly for late than early responding tissues. A simple linear‐quadratic formula can be used to reliably estimate isoeffective doses for different radiation protocols, with *α/β* values representing the tissue‐specific element and with correction for proliferation, if required. These radiobiological principles, encapsulated in the “4Rs” of fractionated radiation therapy,^55^ indicate that the best curative radiotherapy is a balancing act that exploits differences in the growth kinetics of normal tissues and tumor. The impact of these key radiobiological tenets on patient outcome was most clearly demonstrated by several randomized trials studying fractionation for treatment of head and neck cancers during the end of the last century.^56^, ^57^, ^58^, ^59^

In treatment planning for radiation therapy, margins are usually added to create planning target volume (PTV) from gross tumor volume (GTV) and clinical target volume (CTV). This is to account for the setup uncertainty and various voluntary and involuntary tumor motions so that adequate coverage of the tumor will be achieved with the planned treatment.^60^, ^61^, ^62^, ^63^, ^64^ As a result, however, not only tumor but also the surrounding normal tissues falling within the PTV margin receive the prescribed dose. In order to minimize those unintentionally treated normal tissues, higher setup accuracy and better motion management is warranted. Nowadays, the development of the imaging‐guidance (IGRT) and motion management (breath‐hold, gated treatment, tumor tracking, and immobilization) has helped to reduce the margins significantly.^60^, ^63^, ^64^ This, in conjunction with the advancement in intensity‐modulated radiation therapy and volume‐modulated arc therapy (IMRT/VMAT) has enabled hypofractionation in many cancer treatments by reducing the volume of healthy tissues irradiated during treatment. On the other hand, the impulse to decrease margin size in order to allow for further hypofractionation has the potential for peril in the setting of definitive radiation for locally advanced head and neck cancer. Adequate disease control requires sufficient expansion of the treatment volumes around the gross disease to include the areas at risk for microscopic disease. The attempt to decrease clinical target volumes (CTV) and planning target volumes (PTV) in order for to allow for safe delivery of hypofractionated radiation has resulted in a decrease in local regional control.^65^

For some tumor subtypes, such as breast and prostate cancers, that have the characteristic of similar growth kinetics to late responding normal tissue, there has been renewed interest in hypofractionation. Investigation into the risk of late reactions following treatment with modest hypofractionation using modern breast cancer techniques has been encouraging. Even when treatment fields include nodal regions, there does not appear to be an increased risk of brachial plexopathy compared to conventional fractionation.^66^ However, one must look at these data with caution as the median follow‐up time is relatively short at 5.7 years. Although it is generally true that late reactions such as fibrosis occur 6 months to 5 years after treatment, the insidious nature of postradiation nerve damage can result in an extremely long latency period. A retrospective analysis of 150 women who received moderately hypofractionated postmastectomy radiation (44–45 Gy in 3–4 Gy fractions) showed a cumulative incidence of upper extremity paralysis of 25% in the 5‐year survivors, 50% in the 10‐year survivors, and 100% in the 30‐year survivors.^67^, ^68^ These authors conclude: “Doses that seem safe at 5 years can lead to serious complications later.” The 2 Gy dose equivalent that resulted in measurable risk to nerve damage was 60 Gy, with a steep increase in risk over 70 Gy.^68^

While modest hypofractionation of 2.25 Gy per fraction has been shown to provide favorable results in early‐stage (T1‐2N0) glottic cancer, hypofractionation for more advanced H + N cancer can result in extreme morbidity. The laryngeal cartilage is a sensitive structure whose function can be easily impacted by the late effects of radiation with attempts at more aggressive hypofractionation. Recent phase I study on more moderate hypofractionation of 3.5–5 Gy per fraction for early‐stage glottis cancer was closed prematurely due to dose‐limiting toxicities including arytenoid necrosis requiring supraglottic laryngectomy.^69^ This example illustrates the undesirable outcomes that can come from hypofractionation, even in the context of small target volumes and state of the art technology with a stereotactic technique using 3mm CTV/PTV margins, IGRT and VMAT planning.

Similar to the larynx, the pharyngeal muscles and mandible are exquisitely sensitive to late radiation toxicity. Even mild hypofractionation in the setting of a tumor that either invades or is immediately adjacent to the mandible results in unacceptably high rates of osteonecrosis of the mandible.^70^ Early investigation on the effect of fraction size on toxicity following radiation for oropharyngeal cancer provides an estimate that the α/β value for the mandibular bone may be as low as 0.85 Gy (many late responding tissues range in the 2–3 Gy range). This puts the mandible at the lower end of tolerability for increased fraction size and warrants caution when a significant volume of the mandible is inside the treatment field due to intimacy with the target.^71^ The impact of these severe, life‐long toxicities is all the more poignant in the populations who have a high cure rate, such as HPV+ cancer of the oropharynx, as they are living with these effects for decades after curative therapy.

Received wisdom is, of course, rarely universally correct and “habit is a great deadener,” but another round of attempts at hypofractionation that ignore the need for CTV expansions and/or radiobiological considerations would be misguided, and likely dangerous. It may be possible to choose to use hypofractionation for prostate and breast, providing the dose per fraction is not too intense and is delivered with modern techniques that minimizes PTV expansion and optimizes dose homogeneity. Treatment of small lesions, where volume becomes more critical, may be achievable under certain conditions,^55^ but the notion that IMRT or any other current delivery system allows universal application of hypofractionation irrespective of the biology portends years of future major complications for patients. Apart from anything else, radiation oncologists treat patients, not tumor targets, and treatment regimens must be individualized. Differences in growth kinetics between normal tissues and tumor are a major consideration, but many other factors such as tumor volume, dose inhomogeneity, infection, smoking, and prior surgery contribute to the final prescription. Hypofractionation has a place in radiation therapy, but to suggest it could be used without consideration of the biological context is naïve and dangerous.

## 4 Rebuttal

### 4.A Michael Green, MD, PhD; Samantha Van Nest, PhD; Emilie Soisson, PhD (FOR)

We thank our esteemed colleagues for their thoughtful arguments against the adoption of hypofractionated radiotherapy. Indeed, we also believe that “habit is a great deadener,” and it is time to stop defaulting to conventional fractionation due to a belief that prescribing more than 2.2 Gy per day leads to inferior outcomes.

Our colleagues point out that hypofractionation requires tighter margins and suggest there is too great a risk of marginal failure to justify its use. Over the past two decades substantial effort has been made to improve on‐board and in‐room imaging technology in radiation oncology. Advanced imaging systems are now used in most radiation therapy treatments. This drive to implement IMRT and IGRT was motived by the fact that improvements in targeting could result in the delivery of highly conformal dose distributions and reduced PTV (not necessarily CTV) margins. Routine IGRT, that now includes kV imaging, CBCT, MRI, optical tracking, automatic image registration, and a myriad of other tools, was intended to open the door to improve the therapeutic ratio in many disease sites. However, the true clinical benefit of these tools will never be fully realized without challenging our conventional thinking on dose and fractionation. Why invest in all this technology without making an effort to improve tumor control with reduced normal tissue complication?

Conventional thinking has in fact been challenged in many disease sites and led to a change in practice to at least moderate hypofractionation for most disease sites. Our opponents cite concern about the adoption of hypofractionation in head and neck cancer, which accounts for 4% of all cancers in the United States. We concur that extreme hypofractionated regimens are challenging to deliver and poorly tolerated in patients with head and neck cancers. Indeed, the risk of toxicity in patients with an exceptionally high cure rate such as HPV+ oropharynx squamous cell carcinoma is difficult to justify. However, our argument is not that extreme or ultrahypofractionation is necessary for all patients, only that doses of more than 2.2 Gy per day are appropriate for all. Moderate hypofractionated approaches have long been a standard of care in the management of head and neck malignancies.^56^ Given the clinical adoption of hypofractionation in the treatment of central nervous system, head and neck, thoracic, abdominal, extremity, and cutaneous malignancies, we conclude that hypofractionation can be used in all disease subsites.

Our colleagues also point out that there is limited follow‐up with hypofractionated regimens, and that late toxicity must be assessed before wholesale adoption. We ask which other medical or surgical oncologic treatment required even a decade of follow‐up prior to clinical adoption? The 10‐year follow‐up from the Canadian whole breast hypofractionation trials was published in 2010, and there has been no evidence of late fibrosis.^72^ Supporting this, they cite hypofractionation trials from the 1960s in which comprehensive nodal radiotherapy was given without calculating field overlap resulting in toxicity. Thankfully, the ability of modern three‐dimensional planning to avoid organs at risk has increased substantially. We conclude that we have sufficient follow‐up data to support the safe administration of hypofractionation.

Our colleagues’ call for improved radiobiologic understanding refers to using the conventional LQ model and 4 R's of radiobiology to describe cell kill and tumor kinetics following hypofractionation citing literature published “at the end of the last century.” Referencing more recently published data, we have argued in favor of hypofractionation for all patients based on extensive preclinical and clinical radiobiological evidence. The striking clinical outcomes associated with hypofractionation challenges us to consider therapeutic benefits beyond linear‐quadratic cell kill and to more deeply probe the molecular basis for the 4 R's. Evidence suggests hypofractionation could promote a paradigm shift in RT through induction of antitumor immunity.^36^ Hypofractionation has reminded us that our models do not encapsulate all clinically relevant phenomenon. Thus, we argue that we should not restrict the use of hypofractionation based on the need for improved radiobiological reasoning within our limited conventional models, particularly when the clinical observations increasingly show that hypofractionation is a safe approach.

Finally, there is a concern that treating all patients with hypofractionation does not provide individualized care. However, we counter that protracting treatment with standard fractionation is even less patient centered. The adoption of shortened courses of radiation therapy in the management of breast cancer saves 2 weeks of inconvenience for the average patient. Resources should be directed toward establishing individualized treatments informed by both patient and tumor features. To date, clinical implementation of such platforms is limited, but several laboratories are working on individualized biomarker discovery platforms for optimizing RT schedules. Some of these approaches include genomic or spectroscopic based signatures of radiosensitivity as well as *ex* vivo patient‐based models to optimize dosing regimens for radiation‐induced immunogenicity.^73^, ^74^, ^75^ These platforms will be valuable in supporting patient‐centered hypofractionation regimens.

Hypofractionation offers equivalent or improved oncologic outcomes and is well tolerated in terms of both acute and late toxicity. IMRT and IGRT have enabled equivalent or improved tumor control and normal tissue toxicity profile with hypofractionation. Advances in radiation physics and radiation biology support a new standard of clinical care in radiation oncology. The answer is clear: all patients should be treated with hypofractionated radiotherapy.

### 4.B Kathryn Huber, MD, PhD; Yixiang Liao, PhD; William McBride, PhD, DSc (AGAINST)

We agree with Drs. Green, Van Nest, and Soisson that the “transformative improvements” in delivery of radiation therapy through advances in image guidance, target delineation, and motion management have led to “unprecedented potential for safe and effective delivery of high dose radiotherapy.” However, we continue to argue against the universal adoption of hypofractionation for all cancer treatments. This bold movement disregards the diverse radiobiological features of both tumors and the intimate normal tissues that cannot be avoided completely, regardless of the current technological advances. While the arguments presented and the references quoted by our colleagues provide evidence for the advantages of hypofractionation for specific clinical situations, there was no evidence provided to support universal application of hypofractionation to all cancer radiotherapy without causing too much harm.

We also agree with our colleagues that “future clinical trials are needed to realize the potential of improved localization.” As they point out, practice patterns display a disconnection between the choice of PTV and frequency of image guidance.^47^ In addition, there currently remains a lack of evidence to support that PTV reduction in the setting of improved localization will significantly improve outcomes by reducing tumor margins. In fact, this practice may worsen local control and should be done in the setting of a clinical trial. In addition, many tumors substantially intermix with normal tissue or have microscopic disease beyond what can be seen with imaging. No level of improved localization will get beyond the need for inclusion of CTV, making the toxicity from hypofractionation prohibitive in the majority of clinical scenarios.

The suggestion that we “revise the LQ model” is misguided. The model is not the problem. The model is fine and based on a huge amount of hard clinical and preclinical data that simply do not support the application of hypofractionation to all tumors or normal tissues. This is not a “theoretical concern” but an important practical clinical issue. Our opponent's own reference from Hypofractionation Treatment Effects in the Clinic (HyTEC) has shown that LQ model may still approximate the total cell death caused by SBRT/SRS through indirect cell death in certain clinical cases.^76^ In addition, with the exception of a few tumor types, prostate and breast specifically, the majority of tumor types have higher *α/β* than their surrounding normal tissues as demonstrated in the HyTEC papers.^77^, ^78^ In fact, the recent Meta‐Analysis of Radiotherapy in squamous cell Carcinomas of Head and neck (MARCH) has shown that hyperfractionation (the opposite of hypofractionation) radiotherapy presents the greatest benefit in improved overall and progression‐free survival.^56^ The fact that a few human tumors have low *α/β* values and may allow hypofractionation, if IMRT/IGRT is used, simply confirms the validity of the model. It does not encourage its use for all cancer treatments.

Nor can radiation‐induced immunity be recruited to support the general use of hypofractionation. Albeit, this is an exciting time as we are seeing a growing utility of immune therapy in the treatment of cancer; currently, immune checkpoint inhibitors are effective only in the proportion of patients with pre‐existing CD8+ T‐cell antitumor responses.^79^, ^80^ The same is generally true for radiotherapy, given with or without a hypofractionated schedule. In fact, there remains limited evidence that radiotherapy can generate *de novo* tumor immunity or boost pre‐existing immunity sufficiently to affect the outcome. We are still a long way off from understanding and regulating what is now still the observance of an occasional abscopal response.

No matter what additional radiobiological factors are invoked — immunity, reoxygenation, repopulation, etc., the only solid evidence that exists for a radiation protocol that can be safely and effectively applied to all cancers is conventional fractionation, which has been honed over the last century. Certainly there are exceptions where hypofractionation makes sense, but to use it universally is a recipe for disaster and a disservice to our patients.

## CONFLICT OF INTEREST

The authors declare no conflict of interest.
